# Lymph node-biomimetic scaffold boosts CAR-T therapy against solid tumor

**DOI:** 10.1093/nsr/nwae018

**Published:** 2024-01-11

**Authors:** Ziyan Liao, Jie Jiang, Wei Wu, Jiaqi Shi, Yanfang Wang, Yuejun Yao, Tao Sheng, Feng Liu, Wei Liu, Peng Zhao, Feifei Lv, Jie Sun, Hongjun Li, Zhen Gu

**Affiliations:** National Key Laboratory of Advanced Drug Delivery and Release Systems, College of Pharmaceutical Sciences, Zhejiang University, Hangzhou 310058, China; Key Laboratory of Advanced Drug Delivery Systems of Zhejiang Province, College of Pharmaceutical Sciences, Zhejiang University, Hangzhou 310058, China; Liangzhu Laboratory, Zhejiang University, Hangzhou 311121, China; Bone Marrow Transplantation Center of the First Affiliated Hospital and Department of Cell Biology, Zhejiang University School of Medicine, Hangzhou 310058, China; Department of Medical Oncology, The First Affiliated Hospital, School of Medicine, Zhejiang University, Hangzhou 310003, China; National Key Laboratory of Advanced Drug Delivery and Release Systems, College of Pharmaceutical Sciences, Zhejiang University, Hangzhou 310058, China; Key Laboratory of Advanced Drug Delivery Systems of Zhejiang Province, College of Pharmaceutical Sciences, Zhejiang University, Hangzhou 310058, China; Liangzhu Laboratory, Zhejiang University, Hangzhou 311121, China; National Key Laboratory of Advanced Drug Delivery and Release Systems, College of Pharmaceutical Sciences, Zhejiang University, Hangzhou 310058, China; Key Laboratory of Advanced Drug Delivery Systems of Zhejiang Province, College of Pharmaceutical Sciences, Zhejiang University, Hangzhou 310058, China; National Key Laboratory of Advanced Drug Delivery and Release Systems, College of Pharmaceutical Sciences, Zhejiang University, Hangzhou 310058, China; Key Laboratory of Advanced Drug Delivery Systems of Zhejiang Province, College of Pharmaceutical Sciences, Zhejiang University, Hangzhou 310058, China; National Key Laboratory of Advanced Drug Delivery and Release Systems, College of Pharmaceutical Sciences, Zhejiang University, Hangzhou 310058, China; Key Laboratory of Advanced Drug Delivery Systems of Zhejiang Province, College of Pharmaceutical Sciences, Zhejiang University, Hangzhou 310058, China; National Key Laboratory of Advanced Drug Delivery and Release Systems, College of Pharmaceutical Sciences, Zhejiang University, Hangzhou 310058, China; Key Laboratory of Advanced Drug Delivery Systems of Zhejiang Province, College of Pharmaceutical Sciences, Zhejiang University, Hangzhou 310058, China; Liangzhu Laboratory, Zhejiang University, Hangzhou 311121, China; National Key Laboratory of Advanced Drug Delivery and Release Systems, College of Pharmaceutical Sciences, Zhejiang University, Hangzhou 310058, China; Key Laboratory of Advanced Drug Delivery Systems of Zhejiang Province, College of Pharmaceutical Sciences, Zhejiang University, Hangzhou 310058, China; Department of Medical Oncology, The First Affiliated Hospital, School of Medicine, Zhejiang University, Hangzhou 310003, China; Department of Laboratory Medicine, The First Affiliated Hospital, Zhejiang University School of Medicine, Hangzhou 310003, China; Bone Marrow Transplantation Center of the First Affiliated Hospital and Department of Cell Biology, Zhejiang University School of Medicine, Hangzhou 310058, China; National Key Laboratory of Advanced Drug Delivery and Release Systems, College of Pharmaceutical Sciences, Zhejiang University, Hangzhou 310058, China; Key Laboratory of Advanced Drug Delivery Systems of Zhejiang Province, College of Pharmaceutical Sciences, Zhejiang University, Hangzhou 310058, China; Liangzhu Laboratory, Zhejiang University, Hangzhou 311121, China; Department of Hepatobiliary and Pancreatic Surgery, The Second Affiliated Hospital, School of Medicine, Zhejiang University, Hangzhou 310009, China; Jinhua Institute of Zhejiang University, Jinhua 321299, China; National Key Laboratory of Advanced Drug Delivery and Release Systems, College of Pharmaceutical Sciences, Zhejiang University, Hangzhou 310058, China; Key Laboratory of Advanced Drug Delivery Systems of Zhejiang Province, College of Pharmaceutical Sciences, Zhejiang University, Hangzhou 310058, China; Liangzhu Laboratory, Zhejiang University, Hangzhou 311121, China; Jinhua Institute of Zhejiang University, Jinhua 321299, China; MOE Key Laboratory of Macromolecular Synthesis and Functionalization of Ministry of Education, Department of Polymer Science and Engineering, Zhejiang University, Hangzhou 310027, China; Department of General Surgery, Sir Run Run Shaw Hospital, School of Medicine, Zhejiang University, Hangzhou 310016, China

**Keywords:** drug delivery, cell therapy, bioinspired biomaterials, cancer immunotherapy

## Abstract

The limited infiltration and persistence of chimeric antigen receptor (CAR)-T cells is primarily responsible for their treatment deficits in solid tumors. Here, we present a three-dimensional scaffold, inspired by the physiological process of T-cell proliferation in lymph nodes. This scaffold gathers the function of loading, delivery, activation and expansion for CAR-T cells to enhance their therapeutic effects on solid tumors. This porous device is made from poly(lactic-co-glycolic acid) by a microfluidic technique with the modification of T-cell stimulatory signals, including anti-CD3, anti-CD28 antibodies, as well as cytokines. This scaffold fosters a 50-fold CAR-T cell expansion *in vitro* and a 15-fold cell expansion *in vivo*. Particularly, it maintains long-lasting expansion of CAR-T cells for up to 30 days in a cervical tumor model and significantly inhibits the tumor growth. This biomimetic delivery strategy provides a versatile platform of cell delivery and activation for CAR-T cells in treating solid tumors.

## Teaser

An injectable lymph node-biomimetic scaffold that gathered the function of loading, delivery, activation and expansion of chimeric antigen receptor T cells was developed to boost anti-solid tumor treatment.

## INTRODUCTION

Chimeric antigen receptor (CAR)-T cells are altered T cells that can eliminate tumor cells independently of the major histocompatibility complex (MHC) [1–5]. To date, this therapy has shown unprecedented success in patients with hematologic malignancies but limited clinical efficacy has been achieved in solid tumor treatments so far [6–10]. Due to the dense extracellular matrix and abnormal vascular system of solid tumors, limited therapeutic cells could successfully access and infiltrate to the tissues [11–13]. In addition, the immunosuppressive tumor microenvironment (TME) hinders retention and activity of the infiltrated cells [14–16]. To tackle these challenges, various cell-delivery strategies that include assisting CAR-T cells in penetrating solid tumors, implanting and releasing them locally to prolong effectiveness of treatment, and co-delivering cytokines/platelets to reverse the TME have been developed [17–25].

**Figure 1.**
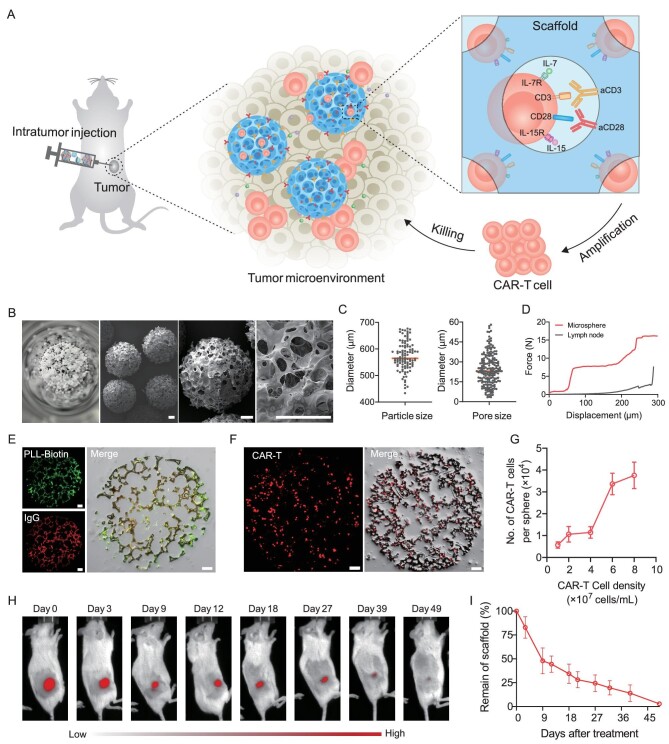
Characterization of scaffold for CAR-T cell delivery and proliferation. (A) Schematic of the artificial lymph node-like scaffold. The scaffold is constructed using the aCD3 and aCD28 antibodies functionalized porous microspheres and provides stimulatory and co-stimulatory signals for T-cell expansion. (B) Representative images of the porous microsphere. Scale bar, 100 μm. (C) Microsphere size and pore size analysis. (D) Mechanical behavior of the scaffold and mouse lymph node. (E) Confocal images of fluorescein isothiocyanate (FITC)-modified PLL-Biotin and rhodamine B-modified IgG-modified porous microspheres. Scale bar, 50 μm. (F) Confocal images of CAR-T cells within the microspheres. Cells were labeled using CFSE. Scale bar, 50 μm. (G) The quantitative flow cytometry analysis of loaded CAR-T cells. *n* = 3, mean ± s.d. (H and I) *In vivo* degradation of the scaffold. (H) The representative fluorescent signal and (I) their quantitative analysis of the Cy5-labeled scaffold after subcutaneous administration. *n* = 5, mean ±* *s.d.

Lymph nodes are small, bean-shaped, encapsulated organs that house a wide range of immune cells and generate multiple immune responses [26–28]. The specific reticular microarchitecture of lymph nodes established by the fibroblastic reticular cells provides favorable mechanical strength for persistent recruitment, survival and spatial organization of immune cells [29,30]. The T-zone, also known as the paracortex is microdomain found in lymph nodes. It is where antigen-presenting cells (APCs) come and display antigens to T cells. The interaction results in rapid stimulation and growth of T cells [31,32]. Herein, we demonstrated a scaffold that mimicked the lymph node environment, including the porous bulk structure in three dimensions and bio-functional stimulatory signals for CAR-T cell delivery. This scaffold gathered the function of loading, delivery, activation and proliferation of CAR-T cells, thereby significantly improving their effectiveness in treating solid tumors (Fig. 1A). In detail, this porous scaffold was made from poly(lactic-co-glycolic acid) (PLGA), a biocompatible polymer, via a microfluidic technique. The average sizes of the pores and the scaffolds were 25 and 570 μm, respectively, thus allowing efficient loading of cells and injection of scaffold directly into tumor sites without surgery. In addition, the scaffold was modified using the T-cell stimulatory anti-CD3 antibodies (aCD3) and co-stimulatory anti-CD28 antibodies (aCD28) signals, as well as cytokines (interleukin-7 and interleukin-15), which mimicked key signal molecules provided by APCs to activate T cells [33–35]. The artificial lymph node-biomimetic scaffold (ALS) demonstrated superior CAR-T cell proliferation both *ex vivo* and *in vivo*. Notably, the injected cells were able to maintain strong killing activity after 50-fold expansion, and the scaffold could support sustained CAR-T cell activation and expansion within tumors for up to 30 days. This biomimetic cell delivery strategy was further demonstrated to be effective at delaying tumor progression when applied to a xenograft tumor model.

## RESULTS

### Characterization of artificial lymph node-like scaffold

The porous polymeric microsphere is a potential cell delivery system to provide sufficient cavity and mechanical force for the loading and retention of cells [36]. Previous research has demonstrated that the microspheres prepared by using microfluidic technology can be adjusted precisely and form a uniform morphological structure compared with those prepared by using the traditional solvent evaporation method [37]. In this work, we fabricated the biodegradable PLGA porous microspheres via microfluidic technology (Fig. S1). The microspheres displayed a spherical morphology and a reticular microarchitecture as observed by using scanning electron microscopy (SEM) imaging (Fig. 1B). They also showed a diameter of ∼570 μm with interconnected pores ranging from 3 to 60 μm in spacing, enabling the potential high loading efficiency of CAR-T cells (Fig. 1C). The microspheres also showed a force of 15 N at a displacement of 300 μm, which protected encapsulated cells and ensured the stability of the scaffold within the tumors (Fig. 1D). The stimulatory antibodies, aCD3 and aCD28, were conjugated to porous microspheres through biotin-streptavidin systems to enhance CAR-T cell proliferation *in vivo* [38]. After treatment by using alkaline hydrolysis (Fig. S2), the biotin-modified poly-L-lysine (PLL-Biotin) was bound to the surface of the microspheres (Fig. S3). To verify the grafting of the antibodies, PLL-Biotin and IgG (as mock antibodies) were modified using fluorescent dyes. The confocal microscopy images of the microspheres (Fig. S4) and their cryo-sections (Fig. 1E) verified the feasibility of the modifications. We also quantitatively calculated the grafting efficiency of the antibodies and the results demonstrated that >90% of the antibodies in the solution could be attached to the microspheres (Fig. S5). The lymph node-like scaffold was assembled by using several functionalized porous microspheres.

We hypothesized that CAR-T cells could enter the interior of the microspheres through micron-sized pores and confirmed the distribution of cells by using confocal microscopy (Fig. 1F). Each microsphere could load >38 000 CAR-T cells when there were 8 × 10^7^ cells per milliliter in the initial cell suspension (Fig. 1G). It is crucial that the functionalized scaffold exists over the duration of the treatment. Thus, to validate the persistence of the scaffold *in vivo*, we developed visualizable microspheres from Cy5-modified PLGA and injected them into NOD.Cg-Prkdc^scid^Il2rg^em1Smoc^ (NSG) mice subcutaneously. The degradation of the scaffold was measured via an *in vivo* imaging system (IVIS). The signals of the scaffold were still detected for 49 days, aligning with the duration of tumor therapy (Fig. 1H and I).

### Evaluation of the influence of the scaffold on CAR-T cell proliferation

**Figure 2.**
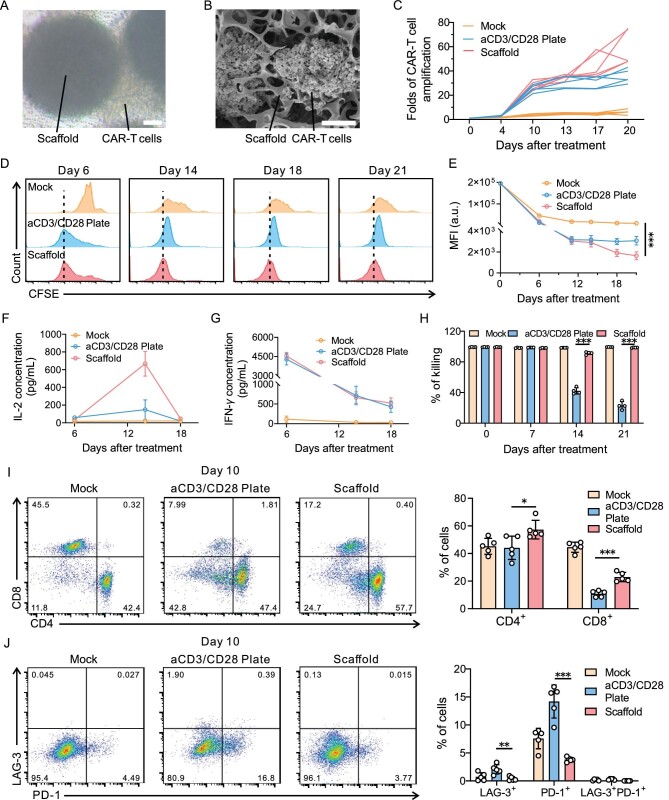
Scaffold promotes CAR-T cell *ex vivo* proliferation and activity. (A) Representative bright-field microscopy images and (B) SEM images of CAR-T cells after 3 days of culture within the scaffold. Scale bar, 50 μm. (C) The expansion curve of CAR-T cells that were untreated (mock), activated with aCD3/CD28 plate or scaffold. (D and E) CFSE-based cell proliferation analysis of CAR-T cells cultured under different conditions. (D) Representative flow cytometry histograms and (E) their relative MFI. *n* = 5, mean ±* *s.d. (F and G) The concentrations of human IL-2 and IFN-*γ* after co-culturing of CAR-T cells with aCD3/CD28 plate or scaffold. Cell number, 5 × 10^5^ CAR-T cells; microsphere number, 50 microspheres. *n* = 5, mean ±* *s.d. (H) The killing activity of CAR-T cells after activation on indicated days. *n* = 4, mean ±* *s.d. (I) Representative flow cytometric plots (left) and relative quantification (right) of CD8^+^ and CD4^+^ CAR-T cells among CD3^+^ CAR-T cells after 10 days of activation. *n* = 5, mean ±* *s.d. (J) Representative flow cytometric plots (left) and the average ratio (right) of PD-1^+^LAG-3^+^ CAR-T cells among CD3^+^ CAR-T cells after 10 days of activation. *n* = 5, mean ±* *s.d. Statistical analysis was performed using one-way ANOVA. *P < 0.05; **P < 0.01; ***P < 0.001.

The CAR-T cells were co-cultured within the scaffold or aCD3- and aCD28-coated cell culture plate (aCD3/CD28 plate) for 3 days. Light microscopy (Fig. 2A) and SEM (Fig. 2B) imaging revealed that high-density clusters between cells and materials were formed, which suggested the activation and proliferation of CAR-T cells [39]. The number of cells was counted during proliferation. As shown in Fig. 2C, the scaffold facilitated a 50-fold total number of cells after activation for 20 days. Besides, the CAR-T cells were marked using carboxyfluorescein succinimidyl ester (CFSE) before co-culturing [40]. Flow cytometry analyses were conducted every week after treatment and the CFSE dilution was observed for 3 weeks, suggesting the sustained cell division (Fig. 2D). By day 21, the mean fluorescence intensity (MFI) values of cells in the scaffold group were only half of those in the aCD3/CD28 plate group (Fig. 2E). We also demonstrated the expansion by using quantitative polymerase chain reaction (qPCR) assays with CAR-specific primers (Fig. S6). The results were further supported by enzyme-linked immunosorbent assay (ELISA) kits. The secretion of inflammatory cytokines interferon-gamma (IFN-*γ*) and interleukin-2 (IL-2)—contributing to CAR-T cell proliferation—was also elevated (Fig. 2F and G). Moreover, the scaffold-activated CAR-T cells maintained enhanced functionality after experiencing improved expansion. The cytotoxicity assay was conducted and demonstrated that CAR-T cells activated by the scaffold showed enhanced antitumor efficacy compared with those activated by aCD3/CD28 plate conditions (Fig. 2H). To figure out how the scaffold affected CAR-T cell properties, we analysed the phenotype and exhaustion markers of the proliferated cells. The percentage of CD8^+^ and CD4^+^ subsets among the CD3^+^ cells in the scaffold group was higher than those in aCD3/CD28 plate group at day 10 and 14, which may have enabled the persistent function of CAR-T cells (Fig. 2I and Fig. S7). Notably, while the CAR-T cells in the scaffold group experienced a rapid expansion, the cells exhibited reduced levels of exhausted markers LAG-3 or/and PD-1 (Fig. 2J). Lactic acid, the degradation product of PLGA, may affect the metabolism of T cells, leading to the changes in the phenotype [41].

### Scaffold serves as a CAR-T cell expansion niche *in vivo*

**Figure 3.**
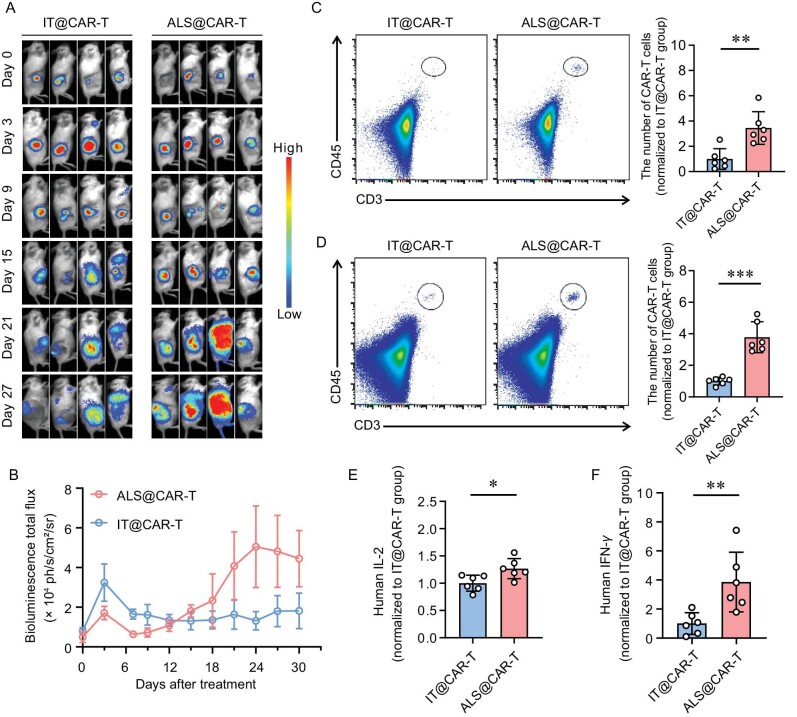
ALS enhances CAR-T cell expansion *in vivo*. (A) The bioluminescence imaging of luciferase-encoding CAR-T cells after injection. (B) Quantitative analysis of the bioluminescence imaging data. *n* = 4, mean ± SEM. (C and D) The representative flow cytometry analysis images (left) and the relative quantified number of CAR-T cells (right). Samples were derived from (C) mouse peripheral blood 21 days post injection and (D) mouse spleen 28 days post injection. *n* = 6, mean ±s.d. (E) Human IL-2 and (F) IFN-*γ* in the peripheral blood 21 days after injection. *n* = 6, mean ± s.d. Statistical analysis was performed using Student's *t*-test. *P < 0.05; **P < 0.01; ***P < 0.001.

We engineered the luciferase-encoding CAR-T cells that could be visible *in vivo* using a bioluminescent imaging method (Fig. S8). Free CAR-T cells or cells delivered by ALS were injected into the subcutaneous tumor sites in NSG mice. The results suggested that, at the initial time points, T cells were released from scaffolds and experienced a slight expansion, but from day 15 to 30, improved T-cell signals were observed (Fig. 3A and B). Ultimately, the CAR-T cells displayed a 15-fold expansion in the ALS-treated mice. After treatment for 3 or 4 weeks, the cells in the peripheral blood or spleens were stained with specific antibodies. Different subsets of cells were identified through such an indicated gating strategy (Fig. S9). The results demonstrated that the number of CAR-T cells in mice treated with ALS@CAR-T is 3-fold higher compared with that treated with free cells (Fig. 3C and D). In addition, we evaluated the concentration of inflammatory cytokines within the mice plasma. The results showing the enhanced concentration of IL-2 and IFN-*γ* in the ALS group were consistent with the quantitative analysis results above (Fig. 3E and F).

### *In vivo* anti-solid tumor activity of ALS@CAR-T

Cytokines such as interleukin-7 and interleukin-15 are considered the third critical factor for the activation of T cells. Thus, we also developed microspheres loaded with these two cytokines to further enhance the therapeutic effects (Fig. S10). Although the half-life of free cytokines is only several hours, the cytokines encapsulated in the microspheres demonstrated sustained retention over 16 days *in vivo* (Fig. S11). The scaffold containing functionalized porous microspheres and cytokine-encapsulated microspheres was used in the subsequent *in vivo* treatment.

**Figure 4.**
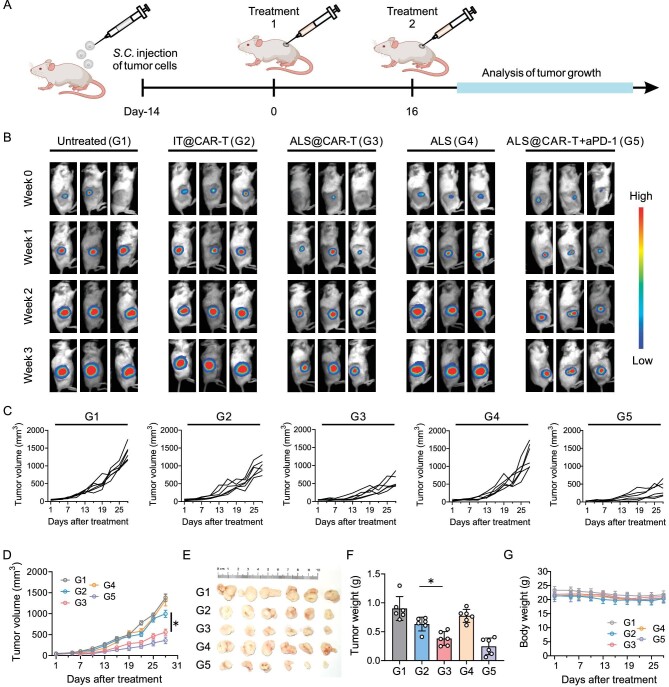
ALS augments CAR-T cell therapeutic efficacy in solid tumors. (A) Schematic of the antitumor study. HeLa cells were inoculated 14 days before treatment. Untreated (G1); IT@CAR-T (G2), CAR-T cells were directly injected into the tumor site; ALS@CAR-T (G3), CAR-T-cell-loaded ALS were intratumorally injected; ALS (G4), only amplified scaffolds were injected into the mice; ALS@CAR-T + aPD-1 (G5), anti-human PD-1 antibodies were applied to assist ALS@CAR-T cells. CAR-T-cell dosage, 2 × 10^6^ cells per mouse; microsphere number, 100 microspheres per mouse. (B) Representative tumor bioluminescence. (C) Analysis of tumor growth. (D) Tumor growth curves. *n* = 6 mice per group, mean ± SEM. (E) Tumors dissected from each group on Day 28. (F) The weight of dissected tumors. *n* = 6 per group, mean ± s.d. (G) Body weight curves after treatment. *n* = 6 per group, mean ± s.d. Statistical analysis was performed using one-way ANOVA. *P < 0.05.

We established a tumor model by subcutaneous injection of luciferase-encoding HeLa cells, which expressed high levels of mesothelin (Fig. S12). When the size of the tumor reached ∼50 mm^3^, the mice were injected with free CAR-T cells (IT@CAR-T) or CAR-T cells loaded in ALS (ALS@CAR-T) intratumorally (Fig. 4A). Mice that were untreated or injected with bare ALS were represented as the controls. Tumor burdens were quantified using IVIS (Fig. 4B). We also plotted the tumor growth curves of each group (Fig. 4C and D). At the end of treatment, the tumors were dissected and weighed (Fig. 4E and F). The results showed that IT@CAR-T showed negligible therapeutic effects in xenograft tumor models, while ALS@CAR-T displayed better tumor rejection. In addition, this therapy can be co-operated with immune checkpoint inhibitors, such as PD-1 antibodies, to synergistically yield increased therapeutic efficiency. No significant difference in the body weight and in the hematoxylin and eosin (H&E)-stained histological section images were found, indicating the negligible toxicity of the materials (Fig. 4G and Fig. S13).

## DISCUSSION

CAR-T cell therapy remains challenging when applied to solid tumors. This is in part because of the dense physical extracellular matrix and the immunosuppressive TME within tumors, which limit the infiltration, inhibit the expansion and cripple the activity of CAR-T cells [42]. To tackle these challenges, some engineered hydrogels or metal thin films are proposed to support the sustained release of therapeutic T cells *in vivo*. The porous microneedle patch aids in breaking down the physical obstacles of tumors, which supports improved locoregional delivery of cells [43,44]. In our work, we drew inspiration from lymph nodes and developed a scaffold to establish an appropriate structural microenvironment for the maintenance of injected cells. The micron-sized scaffold can be injected into tumors without surgery, broadening the application of this system to some inaccessible solid tumors [45]. The scaffold is decorated with stimulatory and co-stimulatory signals, promoting both the delivery and proliferation of cells.

APCs in the specific area of lymph nodes provide T-cell receptor stimulation and costimulation to trigger antigen-specific T-cell activation. Several biomaterials were engineered using T-cell activating cues to boost the rapid proliferation of T cells [46,47]. Commercial microbeads (Dynabeads) are the only FDA-approved devices for T-cell activation *ex vivo* and have become indispensable materials in CAR-T cell manufacturing. However, Dynabeads exhibit significant toxicity to humans and should be separated from CAR-T cells before injection. In our research, the scaffold was also decorated with critical signals of APC (including aCD3 and aCD28), enabling a 50-fold increased *ex vivo* expansion as well as a 15-fold increased expansion of CAR-T cells *in vivo*. We demonstrate that the artificial lymph node-like scaffold can be a safe alternative to Dynabeads and can be injected into patients along with the prepared CAR-T cells without separation. The scaffold can be not only capable of expanding CAR-T cells; it can also be employed as a delivery system to load and reserve CAR-T cells. Moreover, the effects of the scaffold enable a reduced number of initial cells to meet treatment requirement, thereby reducing the manufacturing periods currently required to achieve therapeutic effects. In this study, we demonstrate the therapeutic success in subcutaneous tumors. However, applying the system to other types of cancers, especially to those tumors that are difficult to reach by using surgery, remains to be assessed. Besides of the favorable organ structure and APC, some chemokines and adhesion molecules in lymph nodes are also necessary factors for generating immune responses. So, in order to provide more realistic conditions of lymph nodes, the artificial scaffold can be engineered to present some other functional factors such as CCL21, CCL19, CCR8 and ICAM-1 [48].

Overall, the artificial lymph node-like scaffold serves as a depot for enhancing CAR-T cell expansion and prolonging the efficacy of cells *in vivo* that augments solid tumor treatment efficacy. Furthermore, the preserved ability of the scaffold could be manipulated by coupling microspheres with other materials that convert the immunosuppressive environment within solid tumors.

## MATERIALS AND METHODS

### Fabrication of porous microspheres

A coaxial needle (18 G/25 G) and syringe pump were used to prepare porous microspheres. The emulsion was injected into a syringe as the non-continuous part, which was attached to the inner of the coaxial needle. 1% poly(vinyl alcohol) (PVA) solution was connected to the outer diameter of the coaxial needle, represented as the continuous phase. The two syringes were driven by syringe pumps with a flow rate ratio of 1:20. The microspheres were collected in a beaker containing 100 mL of 2% PVA solution and gently stirred in an ice bath overnight. Then the beaker was moved to a warm bath (45°C) and stirring was continued for 4 hours. The porous microspheres were collected, freeze-dried and stored.

### *In vitro* cell proliferation assay

In total, 500 μL of aCD3 and aCD28 (2 μg/mL) were added to a 24-well plate overnight (4°C) and 5 × 10^5^ CAR-T cells were cultured in the coated plate or in an uncoated plate with 50 amplified microspheres. All samples were cultured in X-VIVO^TM^ 15 medium with cytokines supplementation. Throughout the incubation period, medium was added to maintain the density of cells at <2 × 10^6^ cells/mL. CAR-T cells were counted every week using a blood cell counter (Thermo Fisher) after homogeneous mixing.

The CFSE-labeled CAR-T cells (5 × 10^5^ cells) were cultured using ALS (50 microspheres per well) or in the coated plate. Cytokines (IL-7 and IL-15) were added to the medium. After co-culturing, we collected the cell suspension and the microspheres were placed in 300 μL of phosphate-buffered saline (PBS) four times to release the cells inside. CAR-T cells were harvested from all suspensions by using centrifuge and analysed by using flow cytometry.

Genomic DNA of expanded cells was extracted using a blood genomic DNA miniprep kit (Uelandy). Extracted DNA, *Taq* DNA polymerase and CAR-specific primers (all purchased from Sangon) were mixed and underwent three basic thermal cycling steps of polymerase chain reaction (PCR) (Thermo Fisher).

### *In vivo* antitumor efficacy studies

Once the tumor volume reached ∼50 mm^3^, mice were distributed to several groups randomly and experienced treatment; 2 × 10^6^ free CAR-T cells or ALS loaded with the same amount of CAR-T cells were injected into the tumors via an injection syringe (syringe needle, 1.6 mm). PBS or bare ALS were injected in the same manner to serve as a control group. Some of the mice bearing CAR-T cells delivered by ALS were treated with a combination of 10 μg of anti-human PD-1 antibodies via intravenous injection. The tumor size was monitored using vernier calipers or IVIS every 3 days. At the end of the treatment, the tumors were dissected, weighed and imaged.

## Supplementary Material

nwae018_Supplemental_File
