# Supplementary material for: Lymph node-biomimetic scaffold boosts CAR-T therapy against solid tumor
Source: Natl Sci Rev. 2024 Jan 11;11(4):nwae018. doi: 10.1093/nsr/nwae018 (PMC10911814; doi:10.1093/nsr/nwae018)
Supplement: nwae018_Supplemental_File [file nwae018_supplemental_file.pdf]

Supplementary Materials for

Lymph node-biomimetic scaffold boosts CAR-T therapy against solid tumor

Ziyan Liao[1,2,3]†, Jie Jiang[4]†, Wei Wu[5], Jiaqi Shi[1,2,3], Yanfang Wang[1,2], Yuejun Yao[1,2], Tao Sheng[1,2], Feng Liu[1,2,3], Wei Liu[1,2], Peng Zhao[5], Feifei Lv[6], Jie Sun[4]*, Hongjun Li[1,2,3,7,8]*, Zhen Gu[1,2,3,8,9,10]*

[1]National Key Laboratory of Advanced Drug Delivery and Release Systems, College of Pharmaceutical Sciences, Zhejiang University, Hangzhou 310058, China;

[2]Key Laboratory of Advanced Drug Delivery Systems of Zhejiang Province, College of Pharmaceutical Sciences, Zhejiang University, Hangzhou 310058, China;

[3]Liangzhu Laboratory, Zhejiang University, Hangzhou 311121, China;

[4]Bone Marrow Transplantation Center of the First Affiliated Hospital and Department of Cell Biology, Zhejiang University School of Medicine, Hangzhou 310058, China;

[5]Department of Medical Oncology, The First Affiliated Hospital, School of Medicine, Zhejiang University, Hangzhou 310003, China;

[6]Department of Laboratory Medicine, The First Affiliated Hospital, Zhejiang University School of Medicine, Hangzhou 310003, China;

[7]Department of Hepatobiliary and Pancreatic Surgery, The Second Affiliated Hospital, School of Medicine, Zhejiang University, Hangzhou 310009, China;

[8]Jinhua Institute of Zhejiang University, Jinhua 321299, China;

[9]MOE Key Laboratory of Macromolecular Synthesis and Functionalization of Ministry of Education, Department of Polymer Science and Engineering, Zhejiang University, Hangzhou 310027, China;

[10]Department of General Surgery, Sir Run Run Shaw Hospital, School of Medicine, Zhejiang University, Hangzhou 310016, China

***Corresponding authors.** E-mails: guzhen@zju.edu.cn; hongjun@zju.edu.cn; sunj4@zju.edu.cn

†Equally contributed to this work.

This PDF file includes:

Supplementary text

Fig. S1 to S13

Materials

Poly(D, L-lactide-co-glycolide) (PLGA, lactic: glycolic 75 : 25, Mw = 76,000-115,000, Aladdin), Poly(vinyl alcohol) (PVA, Mw~47000, Aladdin), gelatin (gel strength ≥250 g Bloom, Beyotime), ε-poly-ʟ-lysine•HCl (Mw = 2000-5000, Aladdin), D-biotin N-succinimidyl ester (Aladdin), dichloromethane (DCM), dimethyl sulfoxide (DMSO), triethylamine sodium hydroxide (NaOH) Streptavidin, anti-human CD3 antibodies (Biolegend) and anti-human CD28 antibodies (Biolegend), recombinant human IL-7 (Peprotech) and recombinant human IL-15 (Peprotech).

Emulsion Preparation

Gelatin was dissolved in deionized water and PLGA was dissolved in DCM with a final concentration of 7.5% and 2% (wt%), respectively. To prepare the emulsion, mix the gelatin solution (1 g) and PLGA solution (2.26 g) with the homogenizer (15000 rpm/min) for 3 minutes.

Synthesis of biotin-modified poly- ʟ -lysine (PLL-Biotin)

D-biotin *N*-succinimidyl ester (0.05 g), ε-poly- ʟ -lysine·HCl (0.10 g), and triethylamine (0.13 mL) were dissolved in DMSO (4 mL). 24 hours later, anhydrous ether was added to the reaction solution by drops to form the precipitate. The supernatant was discarded by centrifugation. The product was purified by dialysis in deionized water. The biotin-modified PLL (PLL-Biotin) was collected by freeze-drying. The structure of the product was determined by ^{1}H-NMR.

Antibody-modification of porous microspheres

The porous microspheres were immersed in NaOH solution (0.04 M) for 30 minutes and then washed with deionized water five times. PLL-Biotin was dissolved in deionized water (5 mL) and reacted with microspheres at 4 °C overnight. Then, the streptavidin (50 µg) was added to the washed microspheres and mixed on a thermo shaker at 4 °C for 6 hours.10 µg each of anti-human CD3 antibodies (Biolegend) and anti-human CD28 antibodies (Biolegend) were added and mixed on a thermo shaker at 4 °C overnight after rinsing the microspheres with 5 mL PBS.

To quantitively calculate the grafting efficiency, biotin-modified IgG (Elabscience Biotechnology) were utilized as a mock of the anti-human CD3 and anti-human CD28 antibodies. After incubation, the antibody solutions were collected and added to a streptavidin coated plate (Ruixin Biotech). After one hour of incubation at room temperature, the plate was washed with 0.05% of tween-20. Then, HRP-conjugated affinipure IgG antibodies (Proteintech) were added and incubated. After

wash, the TMB substrate and stop solution were added subsequently. The wells were then read at 450 nm.

Characterization of microspheres

The microspheres were fixed to the scanning electron microscopy (SEM) sample stage by conductive tape and then they were sprayed with gold for one minute. The morphology of microspheres was observed by SEM (Thermo FEI, CZ) and their particle size and pore size were calculated by ImageJ. The modification of microspheres was observed by laser scanning confocal microscope (LSCM, Zeiss, Germany). The mechanical strength of microspheres was assayed by tensile strength tester. A single microsphere was placed in the stainless-steel plate and the force was recorded after deformation.

Cell line

The human cervical cancer cell line HeLa was transduced to express firefly luciferase (FFLuc) and cultured in RPMI 1640 medium (Gibco) supplemented with 10% fetal bovine serum (Gibco) and 1% penicillin-streptomycin (Gibco).

CAR-T cell manufacturing

Human peripheral blood mononuclear cells (PBMCs) were isolated from the peripheral blood of healthy volunteers using human lymphocyte separation medium (Dakewe). Ethical permission was granted by the School of Medicine, Zhejiang University. Informed consent was obtained from all donors. CliniMACS CD3 reagent (Miltenyi) was applied to separate $CD3^+$ T cells from human PBMC. Then the separated $CD3^+$ T cells were activated using TransAct T cell reagent (Miltenyi). By day 2, the activated T cells were infected with CAR-encoding retrovirus or Luciferase-CAR-encoding retrovirus produced from 293T. Infected T cells were expanded for another 8 days to meet the treatment requirements. The expression of CAR in T cell samples was tested by flow cytometry via APC anti-HA.11 epitope tag antibody (Biolegend) staining. CAR-T cells were cultured in X-VIVO™ 15 medium (Lonza) with cytokines, human recombinant interleukin-15 (IL-15, 10 ng/mL) and human recombinant interleukin-7 (IL-7, 5 ng/mL). Cells were incubated at 37 °C in an incubator (Thermo Fisher Scientific) under an atmosphere of 5% CO_2 and 90% relative humidity.

CAR-T cell encapsulation

CAR-T cells were suspended in cell culture medium with concentration with 1, 2, 4, 6, 8 × 10^7 cells/mL. 100 porous microspheres were placed in the cell suspension for 40 mins. Then they were placed into a vacuum environment for 20 mins. The CAR-T cells suspension was discarded, and 300 μL of PBS was added to release the CAR-T cells within the microspheres for 1 hour. Repeat this procedure for four times. All of CAR-T cells in PBS were collected and calculated by flow cytometry with precision count beads (Biolegend). To observe the CAR-T cells loaded within the microspheres, CAR-T cells were incubated with carboxyfluorescein diacetate succinimidyl ester (CellTrace™ CFSE, ThermoFisher) for 15 mins. After 2 days of culture, the CAR-T cells loaded microspheres were freeze-dried to be characterized by the SEM or cut into 10 μm sections. The distribution of CAR-T cells was visualized *via* LSCM.

***In vitro* cell assay**

The cytokines secreted by CAR-T cells in the supernatant were monitored by the enzyme-linked immunosorbent assay (ELISA) kit (Multisciences). To analyze the subtypes of CAR-T cells, the amplified T cells were collected and stained with anti-CD3, anti-CD4, anti-CD8, anti-LAG-3, and anti-PD-1 fluorescent antibodies (All purchased from Biolegend) for flow cytometry analysis.

***In vitro* cytotoxicity assay**

1 × 10^4 luciferase-encoding HeLa tumor cells per well were seeded to 96-well black cell culture plates and cultured overnight. Then, the same number of expanded CAR-T cells were added to the well plates and co-cultured with tumor cells. Tumor cells alone were determined as the control. After 24 hour of incubation, the cell culture supernatant was discarded and 100 μL D-Luciferin potassium salt solution (150 μg/mL) was added. The bioluminescence of retained tumor cells were analyzed immediately by microplate reader (Thermo Fisher).

Scaffold *in vivo* degradation study

1% Cy5-PLGA (Chongqing Yusi) were mixed into PLGA powder while fabricating microspheres. Then, 100 Cy5 marked microspheres were subcutaneously injected into NSG mice. *In Vivo* Imaging Systems were used to detect the fluorescent signals.

Fabrication of IL-7/15 loading microspheres

Recombinant human IL-7 (Peprotech) and IL-15 (Peprotech) lyophilized powder were dissolved in sterile water respectively at a concentration of 220 μg/mL. 50 μL IL-7 and IL-15 solution were

added to 450 μL DCM containing 2% PLGA. Then, the homogenizer was applied to mix the solution and generated the emulsion (8000 rpm/min, 1 min). The emulsion was added drop by drop to the 1% PVA solution under stirring. After 4 hours of stirring, the IL-7/15 loading microspheres were collected and characterized.

Cytokines *in vivo* release study

50 mg of IgG and 0.25 mg of Cy5 were mixed in bicarbonate buffer overnight. Then, the Cy5 labeled IgG (Cy5-IgG) were harvested after dialysis and lyophilization. The microspheres were prepared *via* the mentioned method, wherein the cytokines were replaced with the same amount of Cy5-IgG and were injected into NSG mice subcutaneously. The fluorescence of the microspheres was detected via *In Vivo* Imaging Systems every two days.

Mice and xenograft cancer model generation

All the animals were horsed in the laboratory animal center of Zhejiang University, and all the animal studies were conducted with the approval of their Institutional Animal Care and Use Committee. Six- to eight-week old female NOD.Cg-Prkdc^{scid}Il2rg^{em1Smoc} (NSG) mice were purchased from Shanghai Model Organisms Center Inc. NSG mice are immunodeficient mice established by knockout mutations in the Prkdc (encodes a protein to resolve DNA strand breaks during V(D)J recombination in developing T and B lymphocytes) and Il2rg (encodes the interleukin 2 receptor gamma chain) genes, and are the most immunodeficient instrumental mice available. NSG mice lack mature T cells, B cells, and natural killer (NK) cells. They also lack multiple cytokine signaling pathway and are deficient in innate immunity. 2×10^6 luciferase encoding labelled HeLa cancer cells were inoculated to NSG mice subcutaneously to generate xenograft cancer model.

Flow cytometry

At indicated time point during treatment, 100 μL peripheral blood was collected from the orbit to anticoagulant tubes from treated mice. Then, each tube was added with 1 mL of 1X erythrocyte lysate (Solarbio), followed by incubation on ice for 10 mins, and then centrifuged at 500 g for 5 min. Cell pellets were collected and washed with PBS for twice. Fixable viability stain 510 (BD Biosciences) was then added to stain collected cells at room temperature for 10 mins for distinguishing live cells with dead cells. After washing with PBS, 100 μL cell staining buffer

(Biolegend) was applied to suspend cells and 2 μL Human TruStain FcX™ were added for blocking. Then, the PerCP/Cyanine5.5 anti-human CD45, and Alexa Fluor 488 anti-human CD3 antibodies (all purchased from Biolegend) were added to cells and incubated at room temperature for an hour. The stained cells were analyzed by flow cytometer (Beckman). After treatment, the mouse spleen was dissected and ground. Then the single cells were collected, stained, and analyzed via the same method.

Cytokine release *in vivo*

Peripheral blood was extracted from the mice's eye sockets and collected in EDTA anticoagulant tubes. The plasma was separated through centrifugation (3000 rpm, 10 mins). And the concentration of cytokines was tested by a multiplex cytokine assay kit (Cellgene Biotech).

CAR-T cell *in vivo* proliferation study

1×10^6 luciferase-encoding CAR-T cells were injected into tumor sites directly or through ALS delivery. CAR-T cell proliferation was measured by bioluminescence imaging. At indicated time points, 100 μL of D-Luciferin potassium salt solution (15 mg/mL) was injected into the peritoneal cavity of mice. Six minutes later, the mice were imaged using IVIS and M3Vision software was used to visualize and calculate average luminescence.

Immunohistochemistry

Tumors and other tissues were fixed in 4% paraformaldehyde, dehydrated in EtOH baths, incubated in a paraffin bath, and sectioned to 4 μm thickness slides. After incubating in Hematoxylin and Eosin baths, the sections were observed by VS200 digital slide scanner (Olympus).

Statistical analysis

Unless otherwise stated, data are presented as means ± s.d.. Statistical analysis was evaluated using Prism software (GraphPad). Statistical analysis was performed using one-way analysis of variance (ANOVA) followed by Tukey's post hoc tests for multiple-group analyses and unpaired student's *t*-test for two-group analyses. Results with $P < 0.05$ are regarded as statistically significant differences. Significance is indicated by $^*P < 0.05$, $^{**}P < 0.01$, and $^{***}P < 0.001$ in the figures.

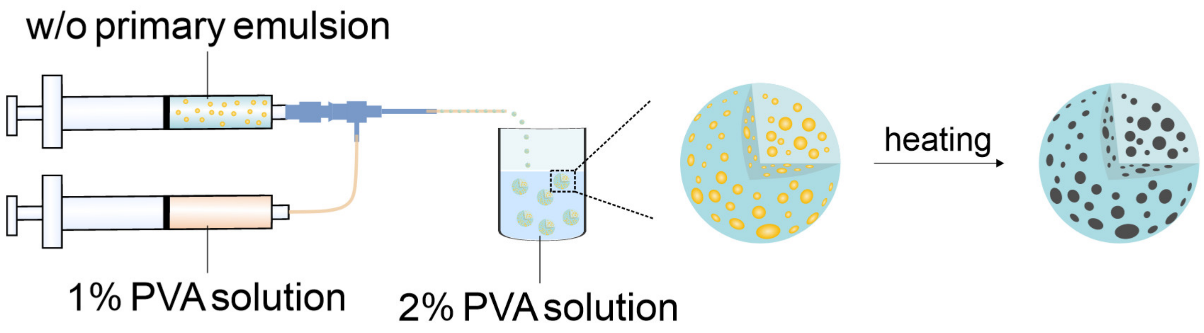

Figure S1. The schematic of microfluidic technology for the production of porous PLGA microspheres.

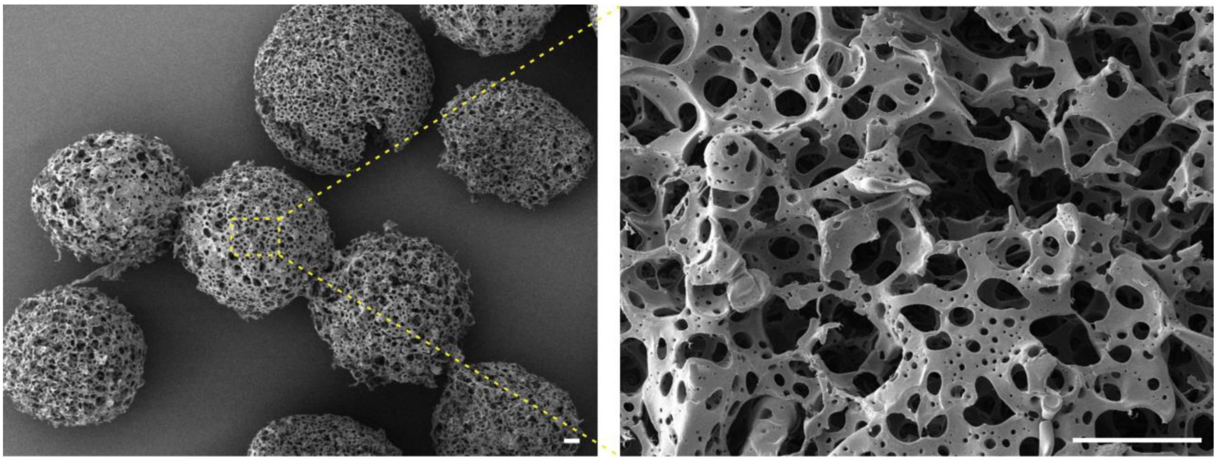

Figure S2. The SEM images of alkaline hydrolysis treated microspheres. Scale bar, 50 μm.

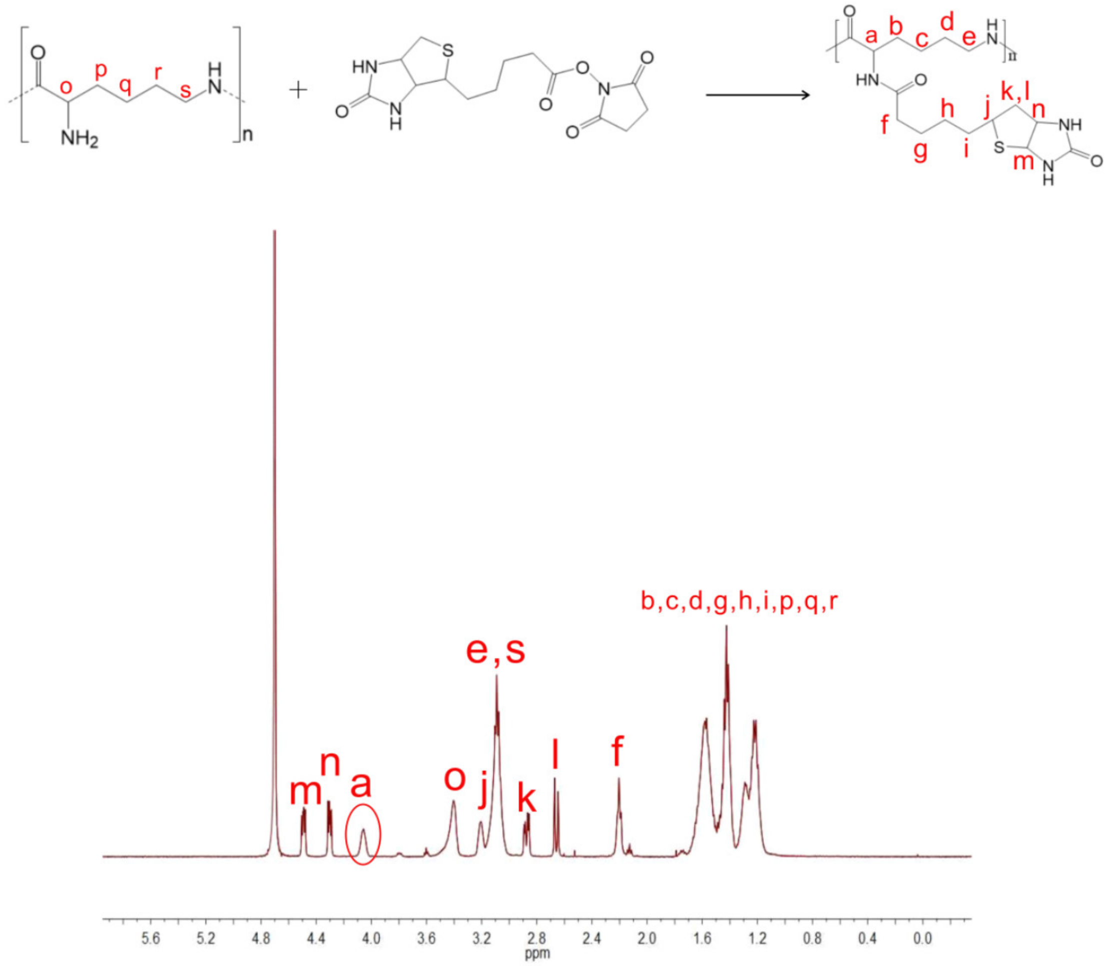

Figure S3. ^{1}H-NMR of synthesized biotin-modified poly- L -lysine (PLL-Biotin).

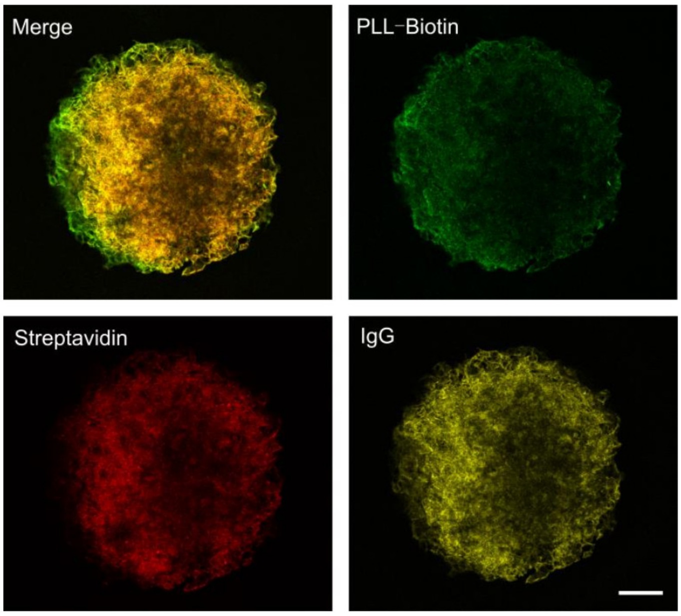

Figure S4. Confocal images of IgG-modified microspheres. PLL-Biotin, Streptavidin, and IgG were labeled with FITC, rhodamine B, and Cy5, respectively. Scale bar, 100 μm.

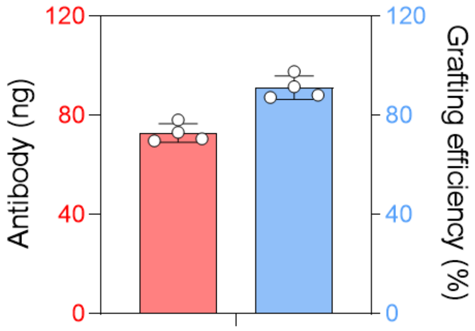

Figure S5. The amounts of antibodies modified to per microsphere and their grafting efficiency. $n = 4$, mean ± s.d.

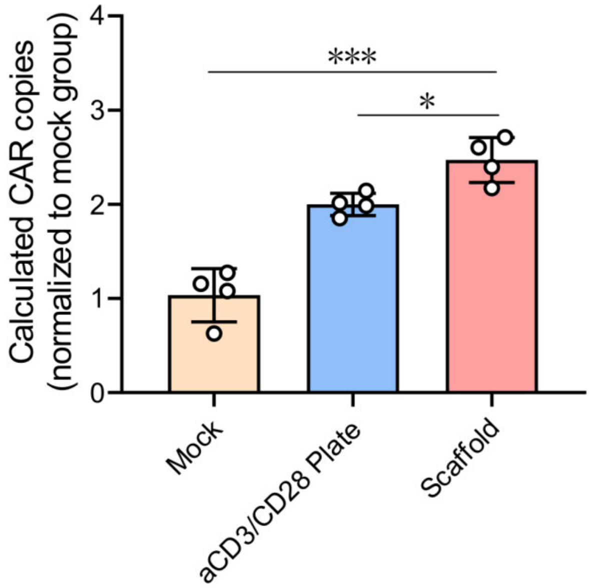

Figure S6. The qPCR analysis of expanded CAR-T cells. $n = 4$, mean ± s.d. Statistical analysis was performed using one-way ANOVA. P value: *$P < 0.05$; **$P < 0.01$; ***$P < 0.001$.

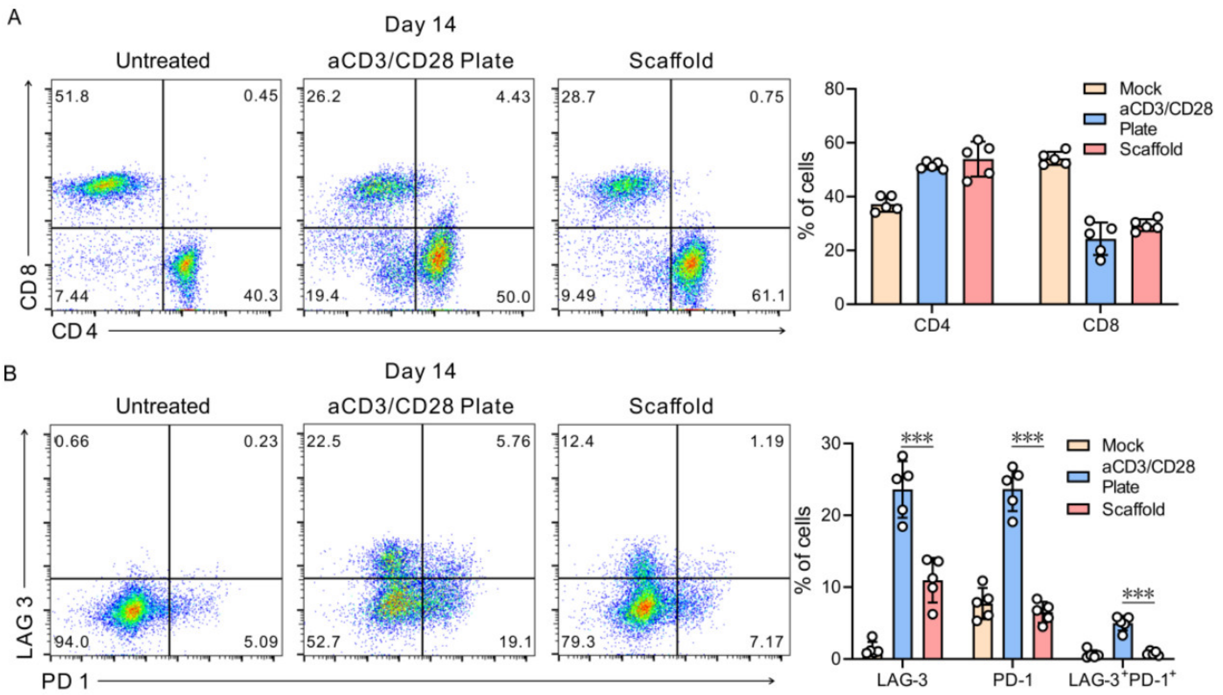

Figure S7. (A) Representative flow cytometric analysis images (left) and relative quantification (right) of $CD8^+$ and $CD4^+$ CAR-T cells among $CD3^+$ CAR-T cells after 14 days of activation. n = 5, mean ± s.d. (B) Representative flow cytometric analysis images (left) and relative quantification (right) of PD-1^+ and LAG-3^+ CAR-T cells among $CD3^+$ CAR-T cells after 14 days of activation. n = 5, mean ± s.d. Statistical analysis was performed using one-way ANOVA. P value: *$P < 0.05$; **$P < 0.01$; ***$P < 0.001$.

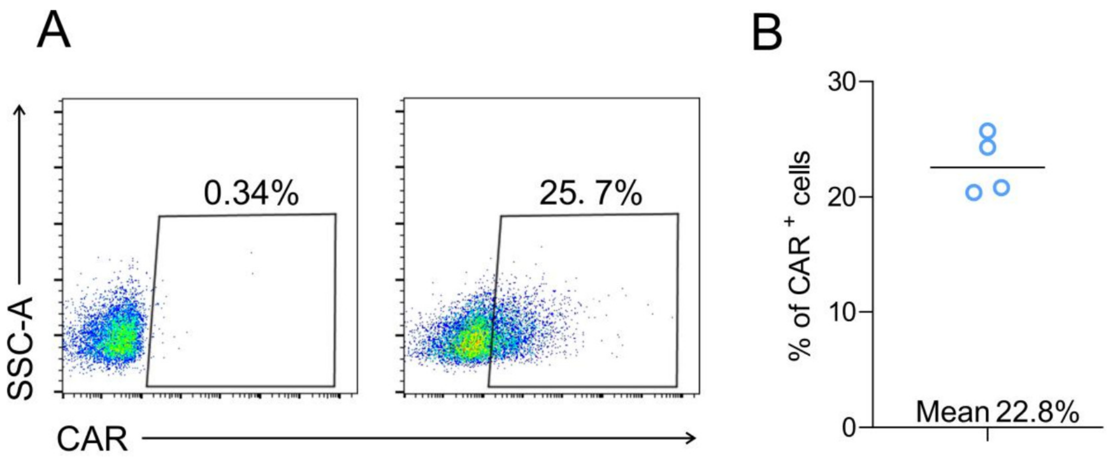

Figure S8. The flow cytometry analysis of CAR expression in T cells after transfection. n = 4, mean ± s.d.

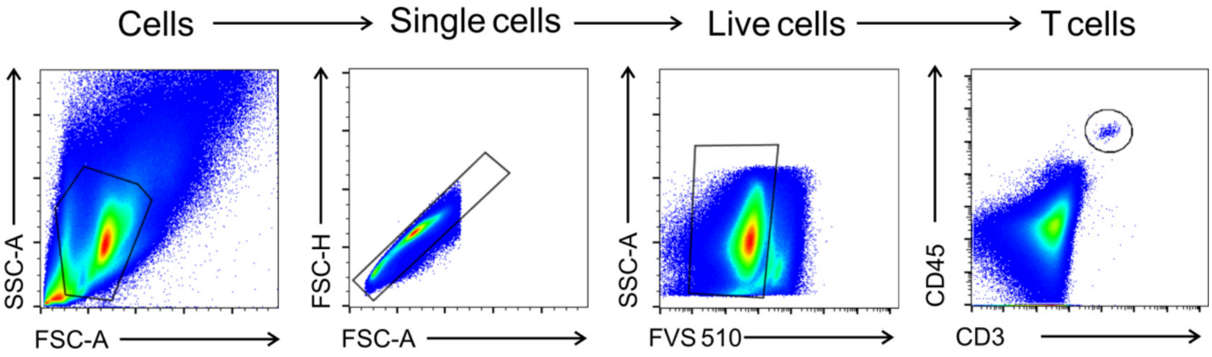

Figure S9. The gating strategy of flow cytometry analysis to recognize T cells *in vivo*.

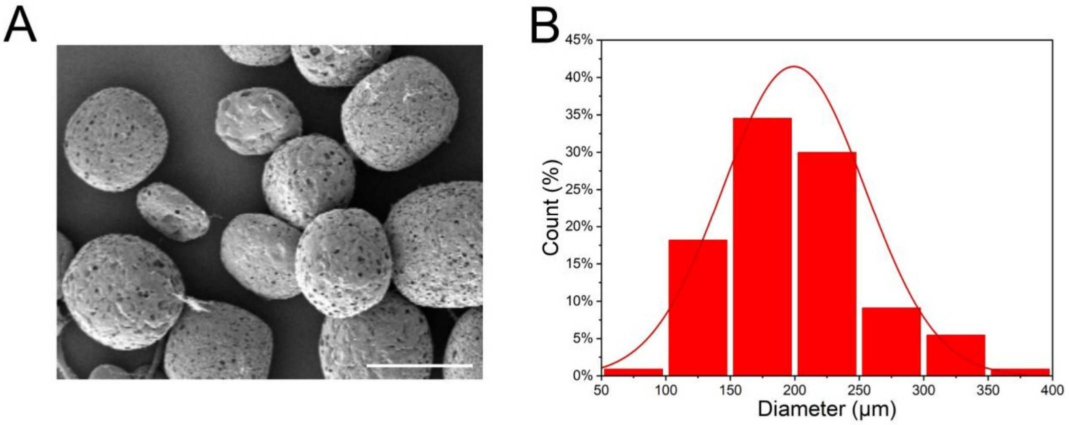

Figure S10. (A) The SEM image of IgG (as a mock of cytokines)-encapsulated microsphere. Scale bar, 200 μm. (B) Distribution of diameters of the microspheres.

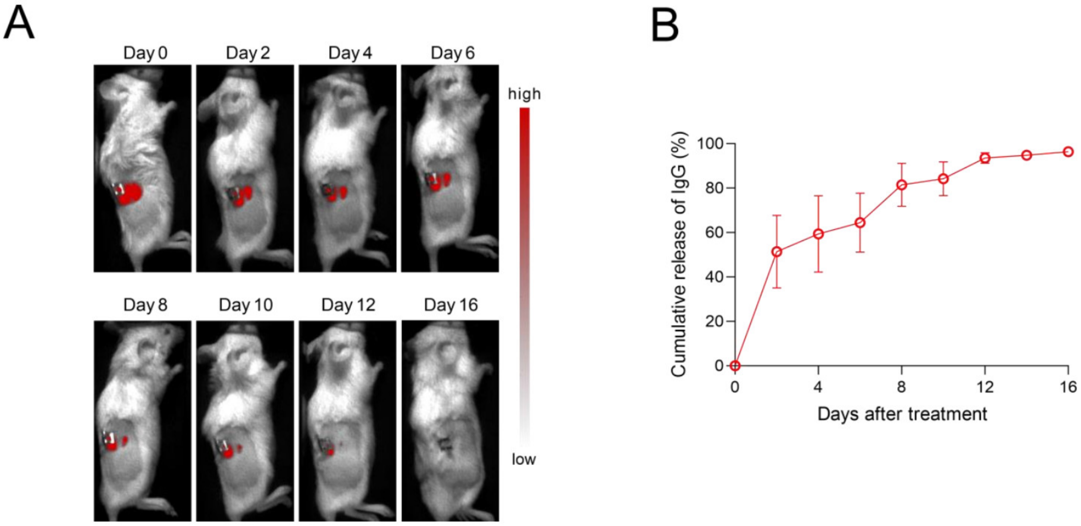

Figure S11. The release of Cy5 labeled IgG from microspheres. The representative fluorescent images (A) and the percentage of cumulative release of IgG (B) were analyzed. $n = 4$, mean ± SEM.

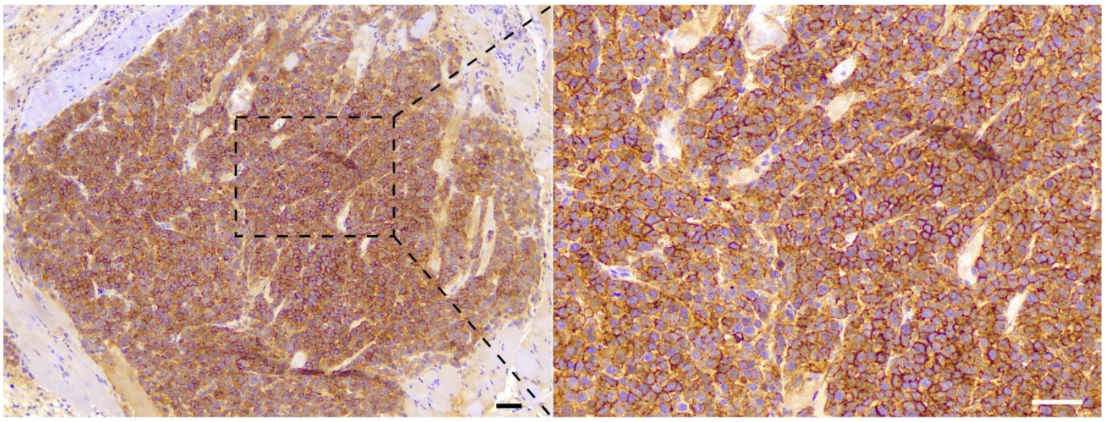

Figure S12. Immunohistochemical staining of mesothelin in HeLa tumors**.** Scale bar, 100 µm.

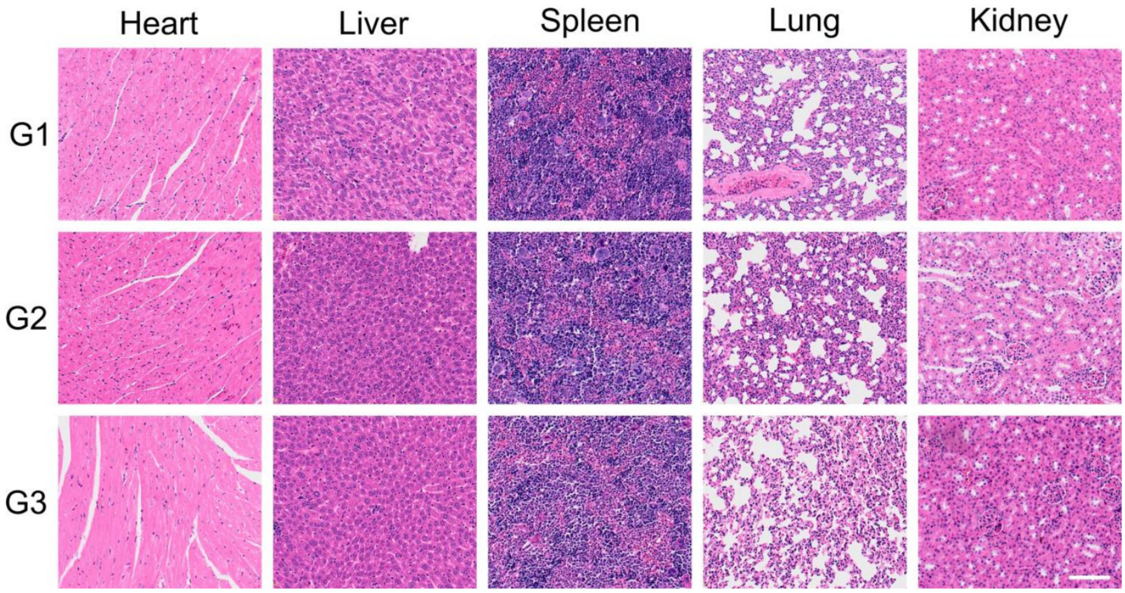

Figure S13. H&E staining sections for heart, liver, spleen, lung, and kidney of mice which were untreated (G1) or treated with IT@CAR-T (G2) or ALS@CAR-T (G3). Scale bar, 100 μm.
